# The Importance of Primary Care Physicians in the Diagnosis and Exclusion of Rare Syndromes: A Case Study of Ruling Out PHACE Syndrome

**DOI:** 10.7759/cureus.51939

**Published:** 2024-01-09

**Authors:** Hannah Lynch, Ezra Dweck, Jehanzeb Nadeem

**Affiliations:** 1 Graduate Medical Education, New York Institute of Technology College of Osteopathic Medicine (NYITCOM), New York, USA; 2 Department of Family Medicine, Jamaica Hospital Medical Center, New York, USA

**Keywords:** interdisciplinary team, infantile hemangioma, pediatric, primary care physicians, follow-up in primary care, phace syndrome

## Abstract

PHACE (posterior fossa malformations, hemangioma, arterial anomalies, coarctation of the aorta/cardiac defects, and eye abnormalities) syndrome is an extremely uncommon condition that requires a team of physicians to properly diagnose, treat, and follow. Patients are at risk of posterior fossa malformations, hemangiomas, and arterial, cardiac, eye, and sternal cleft abnormalities. These syndrome hallmarks can cause severe functional and developmental issues or, in the worst case, death. The pediatrician’s role is even more essential for pediatric patients who present with rare and intricate syndromes. Primary care physicians are the first line of defense for patients. They recognize alarm signs, coordinate patient care, and promote participation. This case report aims to describe the process by which a multisystemic and potentially lethal syndrome was excluded and illustrates the significance of a primary care physician for the diagnosis and management of such conditions.

## Introduction

PHACE (posterior fossa malformations, hemangioma, arterial anomalies, coarctation of the aorta/cardiac defects, and eye abnormalities) syndrome is a rare neurocutaneous condition that affects multiple systems in the body [1]. It affects females more than males with a female-to-male ratio of 9:1 [1]. In 1996, this was coined as a syndrome constituted by posterior fossa malformations, hemangiomas, arterial abnormalities, cardiac complications, and eye abnormalities. Sternal cleft malformations were eventually added to this list and consequently the acronym “PHACE(S)" syndrome was coined [1]. The disease is extremely rare; both its incidence and prevalence have not been established [2]. Even the pathogenesis of the disease has yet to be elucidated. Some theories implicate errors in embryogenesis in the development of PHACE syndrome [1]. 

The exact etiology and genetics of the condition are yet to be revealed [3], but there have been several research studies and case reports over the years to further our understanding. PHACE syndrome has major and minor criteria defined for its official diagnosis [1]. The most commonly reported problems are dermatologic ones, notably infantile hemangiomas (IH) [4], which are required for the diagnosis [1]. There are additional associations as well, including dental abnormalities, headaches, and hearing issues [5]. The arterial abnormalities and cardiac complications, however, pose the most danger to the patient [4]. Although the literature on PHACE syndrome associations is growing, there is still a lack of data for long-term clinical outcomes and prognosis [1]. With varying patient presentations and high-risk complications, it is imperative that PHACE syndrome be diagnosed or excluded as a diagnosis, quickly. 

As the diagnosis is initially suspected based on clinical findings, pediatricians and family medicine providers are usually the first to suspect the possibility of PHACE syndrome. IHs should warrant investigation for the possibility of the disease, as PHACE syndrome accounts for 2-3% of all IHs [4]. The likelihood of PHACE syndrome also increases with both the size and number of hemangiomas, as well as if the IH is present on the face [1,6]. Once PHACE syndrome is suspected, the primary care physician can coordinate care and refer to specialists to rule out associated cardiac, ocular, arterial, and neurological components of the disease.

## Case presentation

**Figure 1:**
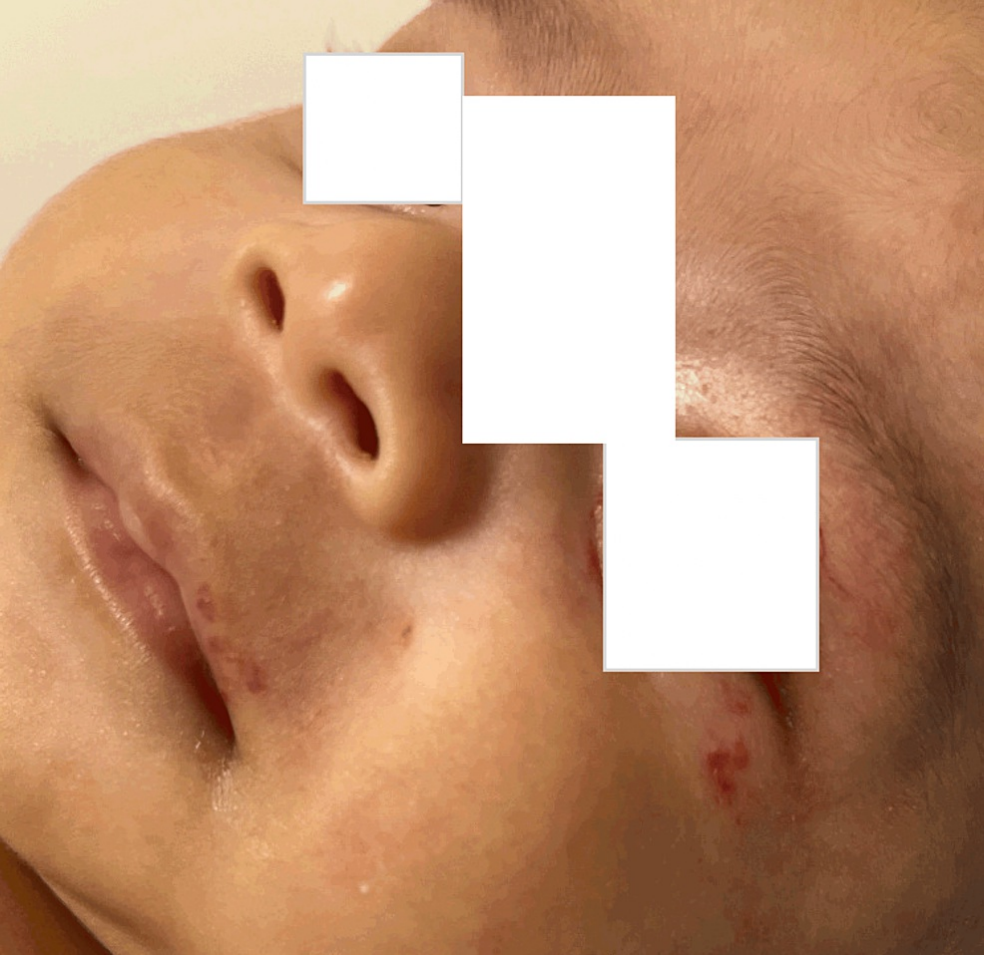
At the age of two months, first diagnosed with hemangioma from left upper lip to left forehead.

A male infant, who was born through a spontaneous vaginal delivery at 2492 grams, presented at a primary care physician’s office at two months with erythematous macules and papules which had coalesced into a plaque, extending from the left forehead to the left upper lip in the V1-V2 distribution. There was an associated conjunctival injection of the left eye, and the left eye also appeared smaller than the right (Figure 1).

These dermatologic findings were not present at birth. At this initial two-month visit, the primary care physician referred the patient to dermatology, who confirmed the diagnosis of IH and recommended extensive workup to rule out PHACE syndrome. Subsequently, the patient was referred to several specialists, including pediatric ophthalmology and dermatopathology, to rule out the diagnosis of PHACE syndrome. The ophthalmic workup yielded no significant findings, and the patient was started on topical timolol twice a day for the hemangioma. This gradually decreased the size of the hemangioma. 

**Figure 2:**
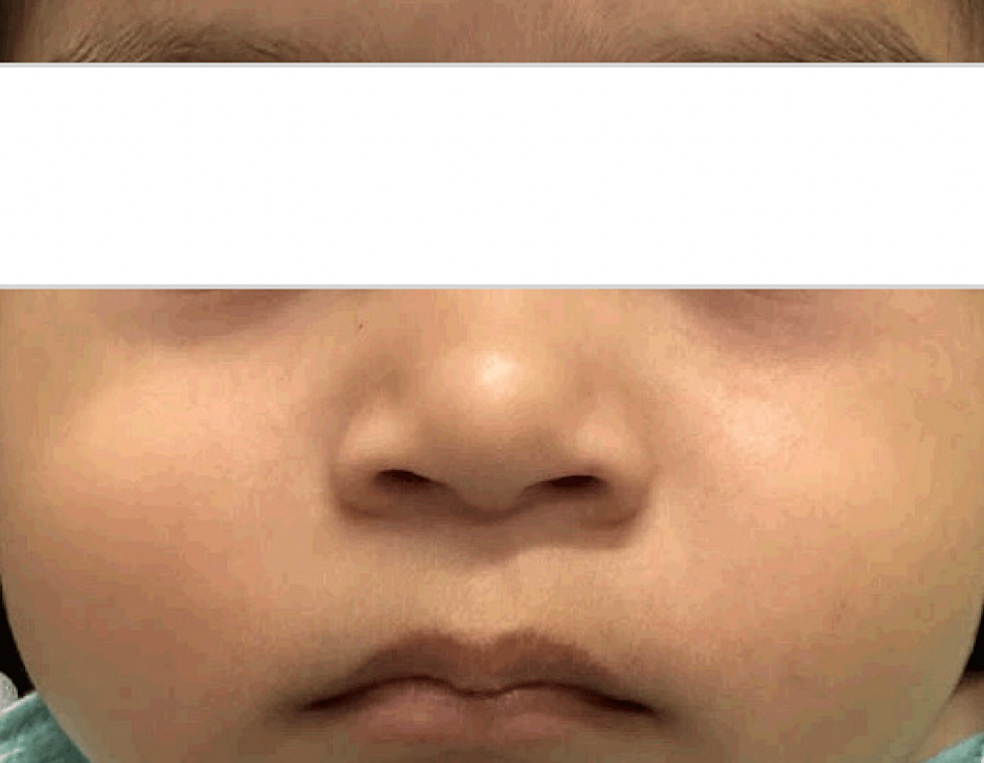
At the age of 13 months, the resolution of hemangioma is seen after using timolol eye drops.

At the six-month visit with the primary care provider, the patient’s mother revealed that she had not seen cardiology or neurology yet and had trouble following up regularly with other specialists due to insurance and travel issues. Also, at this visit, there was concern for gross motor developmental delay as the baby was not meeting appropriate developmental milestones. The appropriate referrals were made again, all within a traveling distance that the patient’s mother was content with. More frequent follow-up appointments with the primary care physician were scheduled to ensure the patient attended all specialist appointments and to monitor the developmental progress of the patient, since specialist appointments can take months to obtain within the hospital’s network. The patient was seen by cardiology and subsequent ECG and Doppler echocardiography studies were within normal limits, with no evident cardiac abnormalities. The patient was seen by neurology which appreciated no focal deficits, and recommended an MRI of the brain at a future visit once the patient was older. By month 13, the patient had full resolution of their hemangioma (Figure 2).

The patient continued to follow up with their primary care physician, and at the 15-month visit, met all developmental milestones. The primary care team will continue to monitor for further developments and a future MRI will be performed to rule out cranial abnormalities. At this time, the diagnosis of PHACE syndrome has been excluded from the differential.

## Discussion

PHACE syndrome is diagnosed by having an infantile hemangioma and either one major or two minor criteria. Major criteria include large brain vessel abnormalities, posterior fossa abnormalities, aortic arch anomaly, ocular posterior segmental anomalies, or a sternal defect [4]. Minor criteria are further complications within the same systems [1]. However, presentation is variable between patients, so suspicion should remain high when evaluating a facial infantile hemangioma. For instance, eye anomalies are found in only 10% of PHACE syndrome cases [7] and so few studies have been done on what specific eye anomalies are common [8]. Since we are still working to understand the syndrome, large infantile hemangiomas on the face should always initiate a PHACE syndrome workup [1-3]. 

Primary care physicians are crucial in diagnosing or excluding the diagnosis of PHACE syndrome and similar rare syndromes. They enable the coordination of care between specialists and act as the first point of contact for patients [9]. Additionally, they maintain a close connection with their patients and are hence able to motivate the patient to receive further care [9]. A visit with a primary care physician is also a frequent route to access specialist care and referrals [9].

For PHACE syndrome workups, a full interdisciplinary team is needed [8]. Our patient had difficulty following up with the various specialists secondary to insurance issues and travel restrictions. Initially, they were lost to follow-up with the specialists but still continued to attend their primary care visits. In the case of our patient, the primary care provider was able to work with the patient to ensure they followed regularly with specialists. The primary care provider assisted the patient in setting up the appointments, referred them to specialists that they were more easily able to access, and met regularly with the patient to monitor progression. Most importantly, the primary care physician was able to elucidate and address any barriers to care.

## Conclusions

The patient in this report presented first for a regular wellness visit in which the primary care team identified the need to rule out PHACE syndrome, a rare and potentially lethal disease, based on the finding of IH. The provider coordinated care with both the patient and specialists to eventually exclude the disease as a possibility. Our case illustrates the integral role of primary care doctors in ruling out multisystemic syndromes.
